# Endoscopic Orbital Clearance/Debridement: A Potential Substitute for Orbital Exenteration in Rhino-orbital Mucormycosis

**DOI:** 10.1055/s-0044-1791645

**Published:** 2025-01-23

**Authors:** Ahmed Hassan Sweed, Ahmed Mohammad Anany, Atef Hussein, Waleed Nada, Mohamed Eesa, Ismail Elnashar, Mohamed Mobashir, Enas Moustafa Ibrahim, Mohammed Elsayed Elmaghawry

**Affiliations:** 1 Department of Otorhinolaryngology, Faculty of Medicine, Zagazig University, Egypt; 2 Department of Ophthalmology, Faculty of Medicine, Zagazig University, Egypt; 3 Department of Diagnostic Radiology, Faculty of Medicine, Zagazig University, Egypt

**Keywords:** COVID-19, mucormycosis, debridement

## Abstract

**Introduction** Mucormycosis is an aggressive, lethal fungal infection affecting the nasal and paranasal territory in immunocompromised patients. Orbital involvement is not uncommon and may require orbital exenteration.

**Objectives** The management of orbital involvement in invasive fungal sinusitis is challenging, ranging from conservative retrobulbar amphotericin B injection in the early stages to orbital exenteration in late stages. Endoscopic endonasal debridement is a minimally invasive technique used to manage orbital fungal involvement in the late stages.

**Methods** Endoscopic endonasal orbital clearance was performed to manage late-stage orbital invasive fungal infection (≥ stage 3c) or after failure of retrobulbar amphotericin B injection with no light perception. Removal of the lamina papyracea (LP) and incision of the periorbita were done to expose all the necrotic intraorbital content in the extra and intraconal spaces. A microdebrider was utilized to debride necrotic fungal infected tissue until a healthy vascularized plane was reached. Gelfoam (Pfizer Inc., New York, NY, United States) soaked in amphotericin B was applied as an adjunctive step to deliver antifungal medication to the orbital content.

**Results** Fourteen patients were included in the study, 9 of whom were male and 5 female, with a mean age of 58.5 years. Eleven patients showed no evidence of disease progression (complete recovery and cessation of medical treatment). Two patients died 15 days after the surgery. The last patient developed frontal lobe abscess but has been treated with double antifungal medication.

**Conclusion** Endoscopic endonasal orbital debridement could be an effective method to treat late-stage orbital fungal infection without jeopardizing the patient's life.

**Level of Evidence** : 4.

## Introduction

Mucormycosis is an aggressive, potentially lethal, angioinvasive fungal infection affecting immunocompromised patients. In the coronavirus disease 2019 (COVID-19) era, the incidence of invasive fungal sinusitis (IFS) increased until evoking a COVID-associated mucormycosis (CAM) health crisis, with high mortality and morbidity. The most well-known risk factors for CAM are immune system dysfunctions, administration of systemic steroids, altered glucose-iron homeostasis, and mechanical ventilation-related problems. 1

Rhino-orbito-cerebral mucormycosis (ROCM) is the most popular form of invasive fungal infection; other forms include pulmonary, cutaneous, gastrointestinal, and disseminated mucormycosis. Rhino-orbito-cerebral mucormycosis is a rapidly progressive disease, even a slight delay in the diagnosis or appropriate management can have devastating implications on patient survival. 2

Early diagnosis and prompt treatment are crucial in the management of ROCM; antifungal medical therapy and surgical debridement until reaching healthy vascularized tissue are applied to all cases. 3

Orbital involvement in ROCM is a miserable condition, which requires orbital exenteration to eradicate it. According to the Code Mucor guidelines for staging, 4 orbital involvement is considered stage 3, further classified into 3a – vision unaffected, nasolacrimal duct and medial orbit involved; 3b – vision unaffected with more than one quadrant or more than two structures involvement; 3c – loss of vision, central retinal artery or ophthalmic artery occlusion or superior ophthalmic vein thrombosis, superior orbital fissure, inferior orbital fissure or orbital apex involvement; and 3d – bilateral orbit involvement. The primary management of ROCM is sinonasal debridement by endoscopic or open approach with proposed protocol for the orbit as follows: Retrobulbar amphotericin B (3.5 mg/mL) is advocated in stages 3a–b, with escalation to orbital exenteration in case of disease progression within 72 hours, and orbital exenteration in stages 3c–d.

A challenge in the management of orbital involvement in ROCM has been the decision to eradicate the disease without orbital exenteration. Orbital exenteration may improve a patient's prognosis but at the cost of a mutilating procedure. Retrobulbar amphotericin B injection may have a role in early orbital involvement, but it currently has no proven effect in the late stages of the condition, and it has questionable effect on patient survival. 2 3 4

In the present study, we assess the feasibility of endoscopic endonasal orbital clearance in late orbital involvement (≥ stage 3c) as an organ preservation protocol instead of traditional orbital exenteration.

## Methods

### Patient Selection and Study Design

This is a prospective case series study conducted at a tertiary care university hospital including 14 ROCM patients with orbital involvement, either in the late stages (≥ stage 3c) (10 cases) or having progressed after retrobulbar amphotericin injection (4 cases).

All affected eyes showed complete ophthalmoplegia, lid ptosis, and total blindness (no perception of light [PL]) (non-serviceable eye), with or/without intracranial involvement.

The current study had institutional review board (IRB) approval ( *n* = 10218/2022), and a signed informed consent form was obtained from all included patients.

We excluded patients with endophthalmitis, or patients with eye lid infiltration/skin necrosis.

### Interventional Aspect

**Fig. 1:**
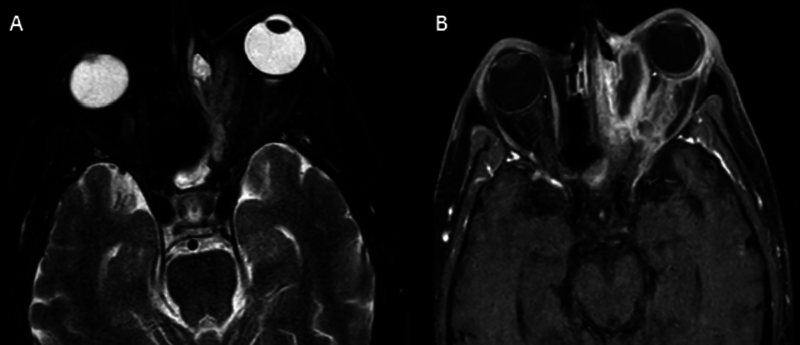
( **A** ) Magnetic resonance imaging T2 FAT SAT axial view shows left proptosis with hyperintense signal within the Naso-Lacrimal Duct (NLD) - medial orbital compartment; ( **B** ) MRI T1 + contrast of the same patient shows left proptosis, loss of contrast enhancement (LOCE) of the medial rectus muscle with LD. enhanced inflammatory reaction around the necrotic muscle.

1- Clinical assessment: All included patients were subjected to ear, nose, and throat (ENT) and ophthalmological examination. The degree of proptosis was assessed in addition to standard ophthalmological data (visual acuity, extraocular muscle [EOM], and ocular fundus examination).
2- As part of the radiological assessment, fat-suppressed T1/ T1c/ T2-sequence magnetic resonance imaging (MRI) scans were requested to identify intraorbital affection (degree of orbital involvement, orbital fat stranding, prominence of superior ophthalmic vein, degree of proptosis) and to identify intracranial involvement (cavernous sinus thrombosis, internal carotid artery [ICA] involvement, brain abscess) 5 ( Figs. 1 2 )
3- Laboratory investigations with serial measurement of erythrocyte sedimentation rate (ESR) and C-reactive protein (CRP).

**Fig. 2:**
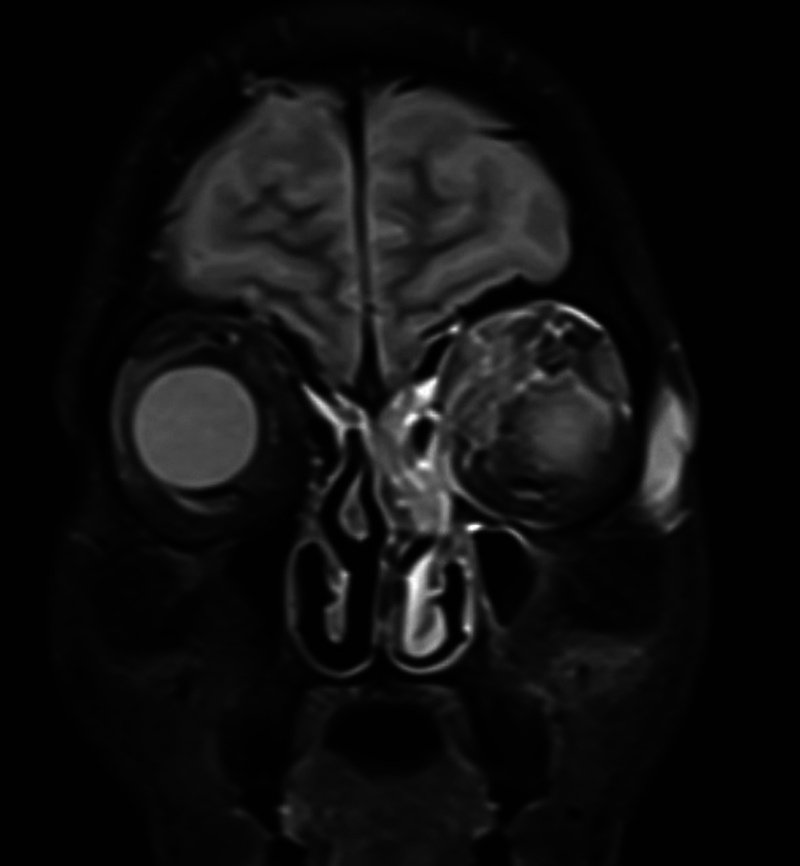
Magnetic resonance imaging T2 fat sat coronal view shows hyperintense signal within Medial superior orbital compartment up to the superior rectus pushing left globe inferolaterally with hyperintense signal within bony lateral orbital margin (zygomatic bone affection) – prominent left ophthalmic vein.

### Surgical Technique

Endoscopic endonasal debridement of fungal-infected and necrotic tissues with nasal cavity and orbit was recommended in the same setting.

We started with a modified Denker approach with sinonasal debridement. Exploration of the posterior wall of the maxillary sinus was done to debride the pterygopalatine fossa (PPF) and infratemporal fossa (ITF) according to disease extension until we reached healthy vascular tissue. Septectomy and other side debridement were achieved in extensive, bilaterally-affected cases.

**Fig. 3:**
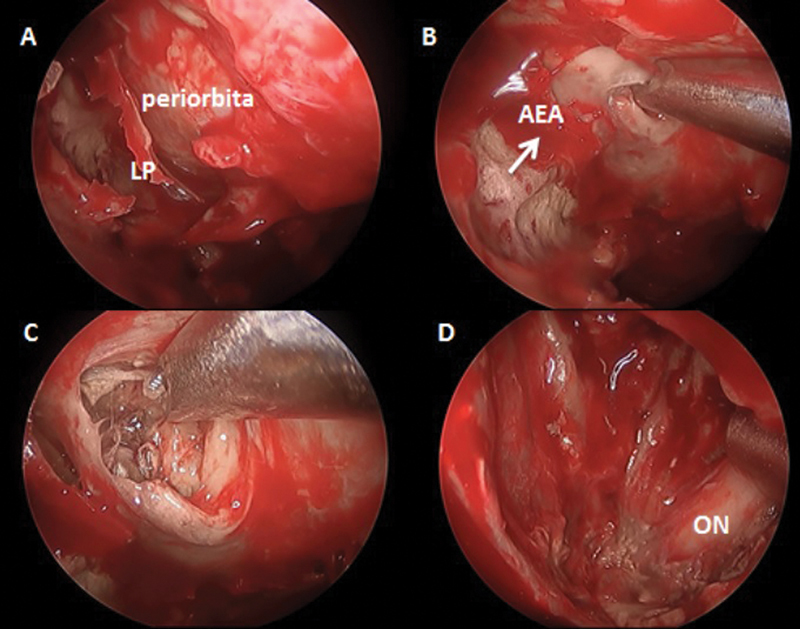
Transnasal endoscopic view of left nasal cavity showing the steps of the procedure. ( **A** ) Removing LP and exposing periorbita. ( **B** ) Opening periorbita with microdebrider, notice the course of AEA which was found totally thrombosed. ( **C** ) Debridement of fungal necrotic tissue. ( **D** ) Final view after reaching healthy vascular tissue with ON exposed but preserved. **Abbreviations:** AEA, anterior ethmoidal artery; LP, lamina papyracea.

Endoscopic endonasal orbital exploration was done by removing the lamina papyracea (LP), incising the periorbita, mobilizing intraorbital-extraconal structures using a ball probe, identifying necrotic intraorbital structures (either fat and/or muscles), and finally removing all these devitalized tissues using a microdebrider ( Fig. 3 ).

**Fig. 4:**
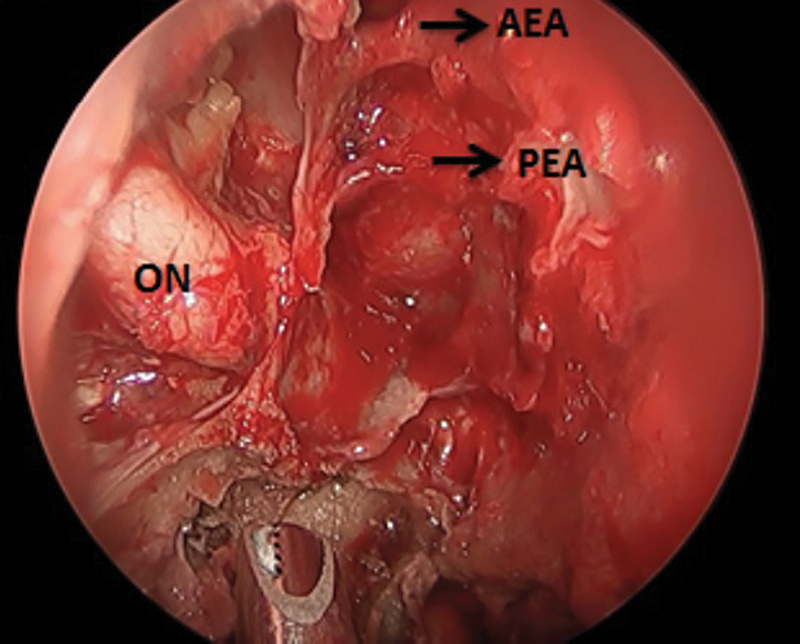
Transnasal endoscopic view of right nasal cavity at the end of the procedure showing evacuated orbital contents with intact ON, notice both AEA and PEA totally thrombosed. Abbreviations: AEA, anterior ethmoidal artery; ON, optic nerve; PEA, posterior ethmoidal artery.

This was done with the aid of a 0-, 30-, and, sometimes, 70-degree scope until we reached healthy vascular tissues, keeping the optic nerve and the eye globe intact. ( Figs. 4 5 )

**Fig. 5:**
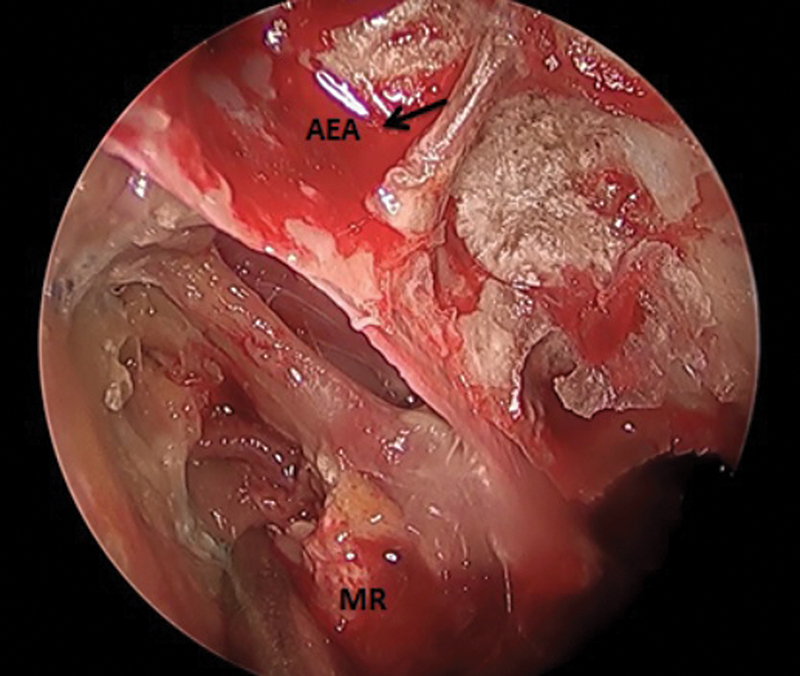
Transnasal endoscopic view of right nasal cavity showing totally thrombosed AEA with severe ischemia and necrosis of the MR muscle and other orbital contents. Abbreviations: AEA, anterior ethmoidal artery; MR, medial rectus.

After complete endoscopic debridement, Gelfoam (Pfizer Inc., New York, NY, United States) soaked in liposomal amphotericin B (LAmB), diluted in distilled water/dextrose 5% (2.5 mg/ml), was applied to all operated areas. The PPF, ITF, orbit, nasal sinuses, and nasal cavity were packed with merocele soaked in LAmB. Nasal packs were removed after 24 to 48 days, after which the diluted amphotericin B in distilled water/dextrose 5% (1 mg/ml) was utilized for nasal irrigation. Endoscopic follow-up was advocated twice a week in the first two weeks of the postoperative period, then weekly in the first month postsurgery, then monthly in the first year.

All patients received standard antifungal treatment (amphotericin B [1–1.5 mg/kg/day] or LAmB [3–5 mg/kg/day]) for 10 to 14 days, with strict follow-up at the hospital to address the side-effects of this drug; this was followed by step-down therapy with posaconazole or voriconazole, according to fungal culture and sensitivity, for an average of 3 to 6 months.

## Results

Fourteen patients were included in the study, 9 of whom were male and 5 female, with a mean age of 58.5 years (29–72). All patients had history of COVID-19 infection prior to development of IFS, and the interval time ranged from 15 to 45 days, with a mean of 21.4 days ( Table 1 ).

**Table 1:** Epidemiology of the included patients (n = 14)

	Age (years)	Sex	Chronic illness	Interval between COVID-19 and IFS (days)	Offending fungus	Degree of proptosis (mm)	ROCM stage	Status	Notes
1	58	M	DM	15	*Mucor*	2.5	Stage 3c	NED	
2	52	M	DM	45	*Mucor*	2	Stage 4a	AWD	Dual antifungal therapy
3	63	M	DM	30	*Mucor*	2	Stage 4b	DOD	
4	56	M	HPT + DM	20	*Mucor*	3	Stage 3c	NED	
5	65	M	DM	15	*Mucor*	2	Stage 3c	NED	Failure of retrobulbar injection (no PL)
6	68	M	DM + HPT + one functioning kidney	15	*Rhizopus*	3	Stage 3c	NED	
7	66	F	DM	20	*Mucor*	1/3	Stage 3d	DOD	LT side endonasal endoscopic orbital clearance RT side – retrobulbar Amphotericin B injection
8	70	M	DM	20	*Aspergillus*	2	Stage 3c	NED	Improved ocular movement
9	55	F	DM, HPT, IHD	21	*Mucor*	2	Stage 3c	NED	Failure of retrobulbar injection
10	54	M	DM	34	*Rhizopus*	3.5	Stage 3c	NED	
11	72	F	DM + HPT	19	*Mucor*	2	Stage 3c	NED	Failure of retrobulbar injection
12	60	M	DM	20	*Mucor*	2	Stage 3c	NED	Failure of retrobulbar injection
13	52	F	DM	27	*Mucor*	3	Stage 3c	NED	
14	29	F	DM	33	*Rhizopus*	4	Stage 3c	NED	Improved ocular movement

**Abbreviations:** AWD, alive with disease; COVID-19, coronavirus disease 2019; DM, diabetes mellitus; DOD; died of disease; HPT, hepatic; IFS, invasive fungal sinusitis; IHD, ischemic heart disease; LT, left side; NED; no evidence of disease; PL, perception of light; ROCM, rhino-orbito-cerebral mucormycosis; RT, right.

In only one patient, the fungal culture revealed the growth of the *Aspergillus* sp. while the *Mucor* and *Rhizopus* spp. were detected in the culture of the remaining patients.

During the follow-up, 2 patients (2/14; 12.2%) died within 15 days of the surgery—one from respiratory failure and the other developed cerebral mucormycosis. One patient had a frontal lobe abscess and was treated with dual antifungal therapy with excellent response to therapy. The remaining patients (11 patients) showed no evidence of disease progression until the writing this manuscript, with average 6-month follow-up period (7 patients had already finished the oral antifungal treatment) ( Table 1 ).

All surviving patients had orbital complaints recurrence—proptosis, lid edema, and conjunctival chemosis—reported within 6 postoperative weeks, during follow-up. There was no improvement in visual acuity and ptosis; only 2 cases showed improvement in ocular mobility (2/12; 16.6%). No patient needed orbital exenteration as a salvage procedure.

## Discussion

Mucormycosis is a potentially life-threatening fungal infection. These fungi are ubiquitous and can be found in fruits, soil, and feces. Fungal spores can infect the oral and nasal cavities through inhalation, but tissue invasion with blood vessel affection (thrombosis), angio-invasion, and tissue necrosis occurs only in immunocompromised patients due to uncontrolled diabetes mellitus, hematologic malignancy, renal failure, acquired immunodeficiency syndrome (AIDS), organ transplantation, among others. 6 7

A multifactorial theory explains fungal superinfection or coinfection in the COVID-19 era either by the occurrence of preexisting diseases, such as diabetes mellitus; use of immunosuppressive drugs, especially corticosteroids; and systemic immune alterations of COVID-19 infection itself, with reduced numbers of T lymphocytes, CD4 T, and CD8 T cells. All these factors may alter innate immunity, leading to secondary and fungal infections. 8 9

After fungal invasion, the process of fungal spread is promoted by direct (tissue angioinvasion) or blood spread. This process leads to orbital and cerebral affection with different degrees. 7

Dealing with orbital mucormycosis is challenging due to both mortality and morbidity issues. Orbital exenteration is the surgical procedure that guarantees disease eradication, but it is a mutilating procedure. Retrobulbar amphotericin-B injection could be effective in controlling orbital mucormycosis without orbital exenteration, but it is only feasible in the early stages. 4

Regarding survival, Choksi et al. 10 revealed that retrobulbar injection decreases the rate of orbital exenteration without reducing the risk of mortality in CAM patients. They added their own thoughts that orbital exenteration cannot increase survival rate. Sen et al. 11 confirmed a significant reduction of mortality in the orbital exenteration group compared with retrobulbar injection (22% versus 33%; *p* = 0.008).

Pathak et al., 12 in their stagewise case series that included 22 patients. Retrobulbar injection had curative role in early orbital involvement (stages 3a–b), but orbital exenteration has to be performed in late orbital involvement cases (stages 3c–3d/4). They found stabilization or slight improvement in visual acuity in early stages, with no effect in patients with no PL. They accounted no improvement at all in ptosis-lagophthalmos central retinal artery occlusion or orbital apex syndrome, non-statistically significant improvement in proptosis, and statistically significant improvement in lid edema and conjunctival chemosis. They confirmed that retrobulbar injection is an effective modality to stop the disease progression in stages 3a, 3b, and/or 3c, but it maybe not be suitable for stages 3d and 4.

Sharifi et al., 13 in his review study including 647 cases with a history of retrobulbar injection(s) of amphotericin B, found that retrobulbar injection can control disease progression, with globe preservation in most cases, with up to 14.9% reduction in the need of orbital exenteration.

Honavar 4 published a protocol-based strategy by a multidisciplinary team to improve ROCM morbidity and mortality. He proposed retrobulbar amphotericin injection in early orbital involvement (stages 3a–b) and orbital exenteration in late orbital involvement. Also, if there disease progression was detected within 72 hours after retrobulbar injection, orbital exenteration should be applied for the sake of disease control.

In the present study, MRI-based surgical intervention was applied, with stress on T2 fat-suppression protocol to find the degree of orbital involvement and other space involvement (PPF-ITF). We tried to find an alternate procedure to orbital exenteration in late orbital involvement (≥ stage 3c) or after failure of retrobulbar amphotericin injection. We started by removing the LP and incising the periorbita, then removing all necrotic tissue with a microdebrider until we reached healthy vascular tissue, keeping the optic nerve and eye globe intact. In all our cases, we could notice that the anterior ethmoidal artery and/or posterior ethmoidal artery were thrombosed, so we could conclude that there is association between both orbital affection and ethmoidal arteries thrombosis. We added Gelfoam soaked with amphotericin B as an adjunctive procedure aimed to deliver medication to all the remaining orbital content.

This technique showed promising results either regarding globe preservation (100%), or ocular symptom (significant improvement in proptosis, lid edema and conjunctival chemosis – 2 patients [2/12; 16.6%] had return ocular mobility – no effect on ptosis) or survival rate (mortality rate [2/14; 14.2%]).

The main limitations of the present study are that it is a single-armed study, without control group, and its small sample size. But due to the nature of ROCM, with high mortality-morbidity burden, a randomized control study is not feasible. Further studies comparing retrobulbar amphotericin B injection, endoscopic endonasal debridement, and orbital exenteration are needed to clarify the management protocol and to customize treatment protocol according to orbital involvement stage to reach best results.

## Conclusion

Endoscopic endonasal orbital debridement with globe preservation could be an effective minimally invasive technique to address orbital affection in ROCM disease in extensive condition or after failure of retrobulbar amphotericin injection.

## References

[1] Sweed A H Mobashir M Elnashar I MRI as a road-map for surgical intervention of acute invasive fungal sinusitis in Covid-19 era Clin Otolaryngol 2021;•••: Epub ahead of print 10.1111/coa.13907 34954903

[2] Mucormycosis ECMM MSG Global Guideline Writing Group Cornely O A Alastruey-Izquierdo A Arenz D Global guideline for the diagnosis and management of mucormycosis: an initiative of the European Confederation of Medical Mycology in cooperation with the Mycoses Study Group Education and Research Consortium Lancet Infect Dis 2019 19 12 e405 e421 31699664 10.1016/S1473-3099(19)30312-3 PMC8559573

[3] Soni K Das A Sharma V Surgical & medical management of ROCM (Rhino-orbito-cerebral mucormycosis) epidemic in COVID-19 era and its outcomes - a tertiary care center experience J Mycol Med 2022 32 02 101238 10.1016/j.mycmed.2021.101238 34979299 PMC8709922

[4] Honavar S G Code Mucor: guidelines for the diagnosis, staging and management of rhino-orbito-cerebral mucormycosis in the setting of COVID-19 Indian J Ophthalmol 2021 69 06 1361 1365 10.4103/ijo.IJO_1165_21 34011699 PMC8302268

[5] Metwally M I Mobashir M Sweed A H Post COVID-19 Head and Neck Mucormycosis: MR Imaging Spectrum and Staging,Academic Radiology,2021, ISSN 1076–6332,https://doi.org/10.1016/j.acra.2021.12.007 10.1016/j.acra.2021.12.007 PMC8673738 34998684

[6] Zhou F Yu T Du R Clinical course and risk factors for mortality of adult inpatients with COVID-19 in Wuhan, China: a retrospective cohort study Lancet 2020 395(10229):1054 1062 32171076 10.1016/S0140-6736(20)30566-3 PMC7270627

[7] Afroze S N Korlepara R Rao G V Madala J Mucormycosis in a diabetic patient: a case report with an insight into its pathophysiology Contemp Clin Dent 2017 8 04 662 666 29326525 10.4103/ccd.ccd_558_17 PMC5754995

[8] Chen N Zhou M Dong X Epidemiological and clinical characteristics of 99 cases of 2019 novel coronavirus pneumonia in Wuhan, China: a descriptive study Lancet 2020 395(10223):507 513 10.1016/S0140-6736(20)30211-7 32007143 PMC7135076

[9] Gangneux J P Bougnoux M E Dannaoui E Cornet M Zahar J R Invasive fungal diseases during COVID-19: We should be prepared J Mycol Med 2020 30 02 100971 10.1016/j.mycmed.2020.100971 32307254 PMC7136887

[10] Choksi T Agrawal A Date P Cumulative mortality and factors associated with outcomes of mucormycosis after COVID-19 at a multispecialty tertiary care center in India JAMA Ophthalmol 2022 140 01 66 72 34882192 10.1001/jamaophthalmol.2021.5201 PMC8662533

[11] members of the Collaborative OPAI-IJO Study on Mucormycosis in COVID-19 (COSMIC) Study Group Sen M Honavar S G Bansal R Epidemiology, clinical profile, management, and outcome of COVID-19-associated rhino-orbital-cerebral mucormycosis in 2826 patients in India - Collaborative OPAI-IJO Study on Mucormycosis in COVID-19 (COSMIC), Report 1 Indian J Ophthalmol 2021 69 07 1670 1692 34156034 10.4103/ijo.IJO_1565_21 PMC8374756

[12] Pathak M Sahu V Arora R D Retrobulbar Amphotericin B Injection in Curbing the Progression of COVID Associated Rhino-orbital Cerebral Mucormycosis: A Retrospective Case Series Indian J Otolaryngol Head Neck Surg 2022 74 02 3352 3358 36246726 10.1007/s12070-022-03199-7 PMC9547996

[13] Sharifi A Akbari Z Shafie'ei M Retrobulbar injection of amphotericin B in patients with COVID-19 associated orbital mucormycosis: a systematic review Ophthalmic Plast Reconstr Surg 2022 38 05 425 432 35943425 10.1097/IOP.0000000000002256 PMC9451608

